# BiRep: A Reputation Scheme to Mitigate the Effects of Black-Hole Nodes in Delay-Tolerant Internet of Vehicles

**DOI:** 10.3390/s21030835

**Published:** 2021-01-27

**Authors:** Catarina Nabais, Paulo Rogério Pereira, Naercio Magaia

**Affiliations:** 1 INESC-ID, Instituto Superior Técnico, Universidade de Lisboa, 1000-029 Lisboa, Portugal; catarina.franca.martins.rolao.nabais@ist.utl.pt; 2 INESC-ID/INOV, Instituto Superior Técnico, Universidade de Lisboa, 1000-029 Lisboa, Portugal; prbp@inesc.pt; 3 LASIGE, Departamento de Informática, Faculdade de Ciências da Universidade de Lisboa, 1749-016 Lisboa, Portugal

**Keywords:** reputation, black-hole attack, delay-tolerant internet of vehicles

## Abstract

Delay-tolerant networking (DTN) enables communication in disruptive scenarios where issues such as sparse and intermittent connectivity, long and variable delays, high latency, high error rates, or no end-to-end connectivity exist. Internet of Vehicles (IoV) is a network of the future in which integration between devices, vehicles, and users will be unlimited and universal, overcoming the heterogeneity of systems, services, applications, and devices. Delay-tolerant internet of vehicles (DT-IoV) is emerging and becoming a popular research topic due to the critical applications that can be realized, such as software or map update dissemination. For an IoV to work efficiently, a degree of cooperation between nodes is necessary to deliver messages to their destinations. However, nodes might misbehave and silently drop messages, also known as a black-hole attack, degrading network performance. Various solutions have been proposed to deal with black-hole nodes, but most are centralized or require each node to meet every other node. This paper proposes a decentralized reputation scheme called BiRep that identifies and punishes black-hole nodes in DT-IoV. BiRep is tested on the Prophet routing protocol. Simulation results show excellent performance in all scenarios, comparable or better to other reputation schemes, significantly increasing the delivery ratio of messages.

## 1. Introduction

Nobody can deny the importance of the internet nowadays. Especially in the times that we are now living, the internet has proven itself fundamental to connect not only communication devices but also us across the Earth. The internet works using a homogeneous set of communication protocols. Network devices that compose the internet use these protocols to communicate with each other, routing data and ensuring message exchanges’ reliability. The usability of the internet depends on various assumptions, but one of the most important is that a continuous bidirectional end-to-end path must be established. What if an end-to-end path is not available? What if the connection establishment time is so long that it is hard to transfer data effectively?

First, introduced to deal with considerable delays and data loss in interplanetary communications, delay-tolerant networks (DTNs) were designed to deal with these challenging scenarios and environments. However, the potential applications on Earth are many. For example, in a natural disaster area, where no end-to-end connection can be established, and internet access fails, the ability to communicate can save lives. In addition, wildlife tracking/monitoring sensor networks, communication in remote and rural areas, developing countries, and vehicular communications are scenarios that benefit from delay-tolerant capabilities [1]. The last application is especially interesting for the real and fast implications it might have on our lives. The Internet of Vehicles (IoV) [2] is a network of the future in which integration between devices, vehicles, and users will be unlimited and universal, overcoming the heterogeneity of systems, services, applications, and devices. Delay-Tolerant Internet of Vehicles (DT-IoV) is emerging and becoming a popular research topic due to the critical applications that can be realized, such as optimizing traffic flow and road capacity, software, and map update dissemination. Moreover, there are also commercial applications such as tourist and leisure information, parking space availability, but most importantly, assisting in communication between emergency services in areas lacking conventional communication means [3]. 

In a DT-IoV network, there is no permanent connectivity with any centralized structure. Nodes can move and communicate by exchanging messages through opportunistic wireless ad hoc connections with other nodes. For a DT-IoV to work and be efficient, cooperation between network nodes is necessary, but it cannot be expected, as nodes might not transmit the messages they receive [4], either because a malicious user controls them, they lack resources, or even because they are selfish and do not want to send messages from other network nodes, only wanting to receive messages for themselves. This latter case is called a black-hole attack. Expressly, the node refuses to transmit any message in which it is not the source and deletes any messages it receives where it is not the destination. Black-hole attacks are one of the most studied attacks in vehicular networks, and although there are already some proposed solutions, none of them are fail-proof or tested for a vast number of scenarios. Throughout the paper, the terms black-hole, malicious, or bad node are used interchangeably to mean simply a black-hole node. 

In this article, the following contributions are presented:

- BiRep is proposed as a new and completely distributed reputation scheme designed to provide an effective and robust identification and punishment of black-hole nodes in a DT-IoV network to diminish their impact on network performance. BiRep can work with any underlying routing protocol.
- The BiRep design is thoroughly described, comprising the evaluation of mechanisms for detecting black-hole nodes, based on message forwarding proofs stored in exchanged messages, the gains achieved by exchanging reputation information with other nodes, and the effect of different punishment actions over black-hole nodes.
- BiRep performance is studied in different scenarios and compared with other related work.

The remainder of this article is as follows: Section 2 presents the background and related work. Section 3 presents assumptions and performance metrics. Section 4 describes the options taken in building BiRep, both for the detection phase, to improve the malicious node detection speed without requiring too many node resources, and for the action phase, to effectively punish malicious nodes without affecting good nodes. Section 5 presents an extensive evaluation of BiRep in different scenarios, assesses the routing performance gains for DT-IoV, and compares BiRep with the related work. Finally, Section 6 concludes and provides ideas for future work.

## 2. Background and Related Work

In this section, a brief description of the theoretical background and related work is given. First, an overview of DT-IoV fundamental concepts is presented. Then, black-hole attacks are presented, and some already existing solutions are presented.

### 2.1. Delay-Tolerant Internet of Vehicles

DT-IoV is an IoV with delay-tolerant capabilities to support long disruptions in network connectivity [2]. This concept was developed considering studies made with Mobile Ad Hoc Networks (MANETs), Vehicular Ad Hoc Networks (VANETs), DTNs, and the Internet of Things. MANETs bring the concept of establishing direct communication between mobile nodes, which do not rely on a fixed infrastructure [5]. VANETs use vehicles as nodes, but contrary to DT-IoV, they assume end-to-end connectivity exists through some path, which can be particularly challenging in high-mobility or very sparse scenarios such as rural or mountainous areas [3]. This is where the DTNs capabilities are brought in as in these environments, the traffic density is usually low, meaning fewer opportunities for contact, and as the vehicles travel at a considerable speed, the contacts will be short. 

VENIAM [6] is a company that is presently already implementing services using the DT-IoV strategy, like mobile data offloading using delay-tolerance. 

### 2.2. Attacks in DT-IoV

DT-IoV routing protocols require a degree of cooperation between nodes belonging to it to deliver their destination messages. However, nodes might misbehave. Nodes’ misbehavior may significantly affect the network performance, as shown in [7,8], a significant problem to be studied in the context of DT-IoV.

One of the most studied attacks is the black-hole attack. The impact on the network that results from this type of attack can vary, affecting the normal functioning of routing protocols in DT-IoV [9]. Not only the impact black-hole attacks can have in a network but also the fact that it is commonly studied within the research community shows how important it is to prevent this attack. This article will focus only on black-hole attacks aiming to identify nodes that perform such attacks and decrease their network effect.

### 2.3. Black-Hole Solutions in DT-IoV

Various mechanisms have been introduced to address black-hole attacks in DT-IoV. One of the first solutions proposed was ferry-based intrusion detection and mitigation (FBIDM) [10]. 

In FBIDM, ferry nodes assist regular nodes in identifying nodes that might be malicious. Ferry nodes circulate the networks, broadcasting messages that good nodes can decrypt. These nodes will exchange with the ferry nodes information about past node meetings and the delivery probability that is used to detect nodes that might be malicious. If a node is declared suspicious a certain number of times, it will be included in the blacklist that ferry nodes broadcast. Blacklisted nodes will be excluded from the network. The FBIDM overall performance is good but is only suited for routing protocols that use information to route, such as PRoPHET [11] or MaxProp [12], as it requires the encounter and delivery information to make decisions. Furthermore, the dependency on the ferry node is a big problem. If the ferry node fails or misbehaves itself, the network becomes compromised. 

A similar solution is presented with the mutual correlation detection scheme (MUTON) [13]. MUTON is similar to FBIDM but considers the transitive property when calculating the delivery probability. Despite the improvements, the problems associated with having ferry nodes persist. 

In [14], the authors proposed a Misbehavior Detection System (MDS) that uses encounter records (ERs), which are similar to encounter tickets. When two nodes meet, an ER is exchanged and used to assess a node’s trustworthiness. The ERs are created after transmitting messages to another vehicular node. The ER includes both node identifiers, a unique sequence number from both vehicles that increases by one after each contact, and a set that identifies the transmitted messages. Every node maintains a Meeting List (ML) and a Local Black List (LBL). The ML stores data from past meetings, like identifiers, meeting time, and trust reputation (TR) from the nodes. ML information can be used to validate the ER later. The LBL stores all malicious nodes locally detected by a node. Nodes will not exchange messages with other nodes in their LBL for some time. To incentivize nodes to cooperate, after the LBL time expires, nodes will be assigned a low TR and removed from the LBL. The MDS system comprises the evaluation module and the decision module. In the evaluation module, vehicular nodes assess the trustworthiness of other vehicular nodes and the TR is updated. The decision module makes the decisions when TRs are received. The MDS achieves high misbehavior node detection rates of over 90% and almost null false-positive rates. Nevertheless, it is important to notice that as trustworthiness is calculated upon contact based on the exchanged ERs, nodes that never meet will not be able to assert each other’s trustworthiness. This means that not all other nodes might detect a malicious node in the network that has few contacts. This can be a problem as, upon a new encounter, a node does not have information about the level of trust that the encountered node has. By contrast, other nodes in the network might already have determined that the node has malicious intent. Moreover, this scheme detects individual attackers well, but it cannot handle the case where attackers collude. This is because a node is considered malicious if it forwards few messages as compared with the messages it receives, which can be measured using ERs. If attackers cooperate, creating valid ERs, malicious behavior can be undetected. 

In [15], the authors extend MDS with cluster analysis, allowing better discrimination between good and malicious behavior, but the problems found in MDS [14] still occur. 

In [16], the authors propose to keep packet delivery records instead of ERs to detect the black-hole attack. A packet delivery record includes identifying the nodes that exchanged packets, the number of received packets from the encountered node, the number of forwarded packets to the encountered node, and the current time-stamp. Each node has two specific tables in its memory, a receiving record table (RRT) and a self record table (SRT). The RRT maintains the most recent packet records generated by its encountered nodes. The SRT keeps the most recent packet records it generates for each node encounter. The scheme begins when two nodes meet. Each node requests the other node’s RRT. With the RRT, a node can calculate if the other node forwards few packets as compared with packets it receives, similarly to the ER scheme of the MDS described above. A threshold is used to mark nodes as suspicious. If the threshold condition is passed, the SRT is used to determine whether the node met drops our packets. When a node is marked as suspicious more than a certain number of times, it is classified as malicious. Otherwise, the nodes exchange packets and generate packet records. This detecting black-holes method has a black-hole detection ratio for different mobility schemes of over 85% and a false-positive ratio of less than 1%. While it can detect packet drops, which [14,15] could not, it still suffers from the same problems. Furthermore, in [16], no punishment mechanism is described to identify malicious nodes or a reward to well-behaved nodes. Without punishment, malicious nodes do not have an incentive to cooperate in the network. 

Another scheme, called RCAR [17], does not detect black-hole attackers but limits the effects of their presence. This scheme also presents some interesting ideas. Identically to previously described schemes, nodes maintain a local notion of reputation. In RCAR, messages carry a list, called nlist, with the identifiers of nodes that forwarded it. If the message passes more than once in the same node, it is not added again. To avoid malicious nodes adding or modifying identifiers, the message also carries a list, called slist, of digital signatures to certify the information in nlist. When a node receives a message, it updates the reputation of nodes that forward the message as specified in the lists. When the message is successfully delivered to its destination, the destination sends an acknowledgment (ACK) message to the sender. The ACK starts with the nlist and slist of the message it is acknowledging. Otherwise, it works as a regular message so that it can follow a different path from the one taken by the original message, and different nodes can contribute to its forwarding, being added to the lists. When the ACK reaches the original sender, it uses the lists’ information to update the reputation information of nodes that forward the messages. Some problems are found in the scheme. Nodes cannot distinguish a message dropped by a black-hole node from a message dropped, because a node has no buffer space. Furthermore, there is no information about the specific node that dropped the message. To manage this situation, an aging mechanism decreases all nodes’ reputation periodically. However, the smaller a node reputation is, the less likely it is that the node is chosen to forward messages. Other problems can be found in RCAR. Namely, not knowing or not having an idea of which node misbehaved, messages from that node can still be received, and the node has no incentive to cooperate. 

In [18], a cooperative watchdog system (CWS) is proposed. The CWS monitors messages delivered, relayed, and dropped. The CWS of a node exchanges reputation information with other nodes, which is a significant advantage as compared with previously described schemes. However, while the impact on routing performance is assessed, the misbehaving detection ratio is not assessed, and neither are the ratios of correct and incorrect classifications. 

Other methods exist to try to solve black-hole attacks in delay-tolerant networks. This section only presented some of them and described only the most relevant parts of those schemes. Table 1 summarizes the main characteristics of the described mechanisms. More comprehensive surveys of solutions to black-hole or other attacks in similar situations may be found in [19,20]. For VANETs, a survey is available in [21]. Most solutions for VANETs assume a centralized authority or cloud server that is always available, generally unconstrained in terms of communication, computation, and storage, which cannot be compromised [22], and thus does not apply to a delay-tolerant scenario that requires a completely distributed solution. 

The main motivation to try to solve the problem of black-hole nodes was the fact that most effective protocols use a reputation value as a base for judgment, the dependency of other nodes for information, and the fact that to classify a node in most reputation schemes, there must be an encounter, not allowing for a good network awareness. A wrong reputation value is problematic in the case you want to punish nodes for malicious behavior. Nodes, good or bad, might be in a limbo of classification before reaching a certain threshold. Furthermore, the possibility that good nodes might have to be continually proving they are good may slow down message exchanges. The dependency on other nodes’ information is a clear drawback when analyzing scenarios with black-holes, having to meet a node before being able to classify it is a drawback. In that way, a node will never be able to identify all the existing malicious nodes in a large scenario. Having these primary three factors in mind, an attempt was made to try to create a reputation system that corrects these drawbacks and also achieves a high black-hole node detection ratio and a 0% false-positive ratio.

## 3. Preliminaries

### 3.1. Assumptions

Several assumptions are made following some aspects of DT-IoV in general. Nodes can move and communicate only by exchanging messages wirelessly with other nodes within radio range. The wireless link can be broken, for instance, if the node in contact goes beyond radio range, which results in an incomplete message transmission being dropped. 

It is assumed that every node has a unique identifier that allows other nodes to distinguish them in the network. Besides, messages carry the list of the nodes’ identifications they have passed by. Starting with the source, each node adds its signed identification upon receiving a message that is not destined to itself, as in RCAR [17]. Moreover, it is assumed that nodes, when receiving a message, can know its source. 

The proposed detection schemes only focus on simple black-hole attacks. This means that a black-hole node maintains its behavior during the whole simulation and does not collude with other black-hole attackers. 

### 3.2. Reputation System Performance Metrics

Before conducting a comparative analysis of the reputation systems, it is essential to clarify the performance metrics used. 

The metrics are divided into two groups. The first is for purposes of evaluating the routing protocols’ performance. The second is to evaluate the node’s classification of each other. In this article, additional metrics are used in both groups. These serve to measure the same metrics but only concerning the good nodes in the network.

#### 3.2.1. Routing Protocol Metrics

Routing protocol metrics aim to measure how well the routing protocols perform when faced with various percentages of black-hole nodes.

The delivery ratio indicates the successfully delivered messages from all the messages that were sent as in:

(1) $$delivery\;ratio=\frac{\mathrm{number}\;\mathrm{of}\;\mathrm{delivered}\;\mathrm{messages}}{\mathrm{number}\;\mathrm{of}\;\mathrm{created}\;\mathrm{messages}}$$

Notice that although in the ONE simulator [23], the same message can be delivered more than once to the recipient, only the first time is accounted for in the equation.

Delivery Ratio for Good Nodes is essentially the same as the delivery ratio. However, only the delivered and created messages from good nodes to good nodes are accounted for, as in:

(2) $$delivery\;ratio\;for\;good\;nodes=\frac{\mathrm{number}\;\mathrm{of}\;\mathrm{delivered}\;\mathrm{messages}\;\mathrm{from}\;\mathrm{good}\;\mathrm{nodes}\;\mathrm{to}\;\mathrm{good}\;\mathrm{nodes}}{\mathrm{number}\;\mathrm{of}\;\mathrm{created}\;\mathrm{messages}\;\mathrm{from}\;\mathrm{good}\;\mathrm{nodes}\;\mathrm{to}\;\mathrm{good}\;\mathrm{nodes}}$$

The latency of a message is the time delay between the creation and the delivery of the message. In the Average Latency for Good Nodes metric, only messages from good nodes to good nodes are accounted for as in:

(3) $$latency=\frac{\sum_{i=0}^{\mathrm{number}\;\mathrm{of}\;\mathrm{delivered}\;\mathrm{messages}}{(\mathrm{delivery}\;\mathrm{time}}_{i}-{\mathrm{creation}\;\mathrm{time}}_{i})}{\mathrm{number}\;\mathrm{of}\;\mathrm{delivered}\;\mathrm{messages}}$$

The overhead ratio of a protocol indicates the excess of messages successfully transmitted compared to the total number of messages delivered. In the Overhead Ratio for Good Nodes metric, only messages from good nodes to good nodes are regarded, as shown in:

(4) $$overhead\;ratio\;for\;good\;nodes=\;\frac{{\;t}_{\mathrm{from}\;\mathrm{good}\;\mathrm{nodes}\;\mathrm{to}\;\mathrm{good}\;\mathrm{nodes}}{-d}_{\mathrm{from}\;\mathrm{good}\;\mathrm{nodes}\;\mathrm{to}\;\mathrm{good}\;\mathrm{nodes}}}{d_{\mathrm{from}\;\mathrm{good}\;\mathrm{nodes}\;\mathrm{to}\;\mathrm{good}\;\mathrm{nodes}}}$$

where *t* represents the number of transmitted messages and *d* the number of delivered messages. 

#### 3.2.2. Node Classification Metrics

The objective of node classification metrics is to measure how well and how fast nodes classify each other as good or malicious in the simulation when faced with various percentages of black-hole nodes. 

The detection ratio represents the percentage of malicious nodes detected by all nodes in the simulation. This means that for a detection rate of 100%, both good and malicious nodes must identify all black-hole nodes. Thus, if the simulation has *B* black-hole nodes, each good node should identify *B* black-hole nodes, and each malicious node should identify *B*-1 black-hole nodes, not counting itself. Considering *BHs* as an abbreviation of black-hole nodes, *N* as the total of good nodes, *B* as the total of black-hole nodes, and *T* as the total number of nodes in the simulation, the detection ratio is calculated using:

(5) $$detection\;ratio\;\left[\%\right]=\frac{\sum_{i=0}^{T}{(\mathrm{number}\;\mathrm{of}\;\mathrm{BHs}\;\mathrm{classified}\;\mathrm{as}\;\mathrm{BHs}}_{i})}{N\times B+B\times (B-1)}\times 100$$

The detection ratio for good nodes is the percentage of malicious nodes detected by good nodes in the simulation. Here, contrary to the detection ratio, only the classification of black-hole nodes by good nodes is relevant. The distinction is made between the two detection ratios because it is in a reputation system. However, all nodes must identify the threats, which is more valuable for the network if all good nodes identify all bad nodes. The detection ratio for good nodes presents a clearer view of this point. Considering *N* as the total of good nodes and *B* as the total of black-hole nodes, this metric is calculated as:

(6) $$detection\;ratio\;for\;good\;nodes\;\left[\%\right]=\frac{\sum_{i=0}^{N}{(\mathrm{number}\;\mathrm{of}\;\mathrm{BHs}\;\mathrm{classified}\;\mathrm{as}\;\mathrm{BHs}}_{i})}{N\times B}\times 100$$

The false-negative ratio is the percentage of black-hole nodes mistakenly classified as good. With a high false-negative ratio, good nodes exchange messages with malicious nodes thinking they are good and cooperate in the message exchange. Considering *T* as the total number of nodes in the simulation, the false-negative ratio is obtained using:

(7) $$false-negative\;ratio\;\left[\%\right]=\frac{\sum_{i=0}^{T}{\left(\mathrm{number}\;\mathrm{of}\;\mathrm{black}-\mathrm{hole}\;\mathrm{nodes}\;\mathrm{classified}\;\mathrm{as}\;\mathrm{good}\right)}_{i}}{\mathrm{total}\;\mathrm{number}\;\mathrm{of}\;\mathrm{classifications}}\times 100$$

The false-negative ratio for good nodes is the percentage of black-hole nodes mistakenly classified as good by good nodes. Considering *G* as the total number of good nodes in the simulation and *BHs* as the number of black-hole nodes, the false-negative ratio for good nodes is obtained using:

(8) $$false-negative\;ratio\;for\;good\;nodes\;\left[\%\right]=\frac{\sum_{i=0}^{G}{\left(\mathrm{BHs}\;\mathrm{classified}\;\mathrm{as}\;\mathrm{good}\;\mathrm{by}\;\mathrm{good}\;\mathrm{nodes}\right)}_{i}}{\mathrm{total}\;\mathrm{number}\;\mathrm{of}\;\mathrm{classifications}\;\mathrm{by}\;\mathrm{good}\;\mathrm{nodes}}\times 100$$

The false-positive ratio is the percentage of good nodes mistakenly classified as malicious. This is a crucial metric if it is decided to punish bad behavior. If the false-positive ratio is high, good nodes may be prevented from contacting other nodes, unfairly impairing the exchange of messages. Considering *T* as the total number of nodes in the simulation, the false-positive ratio is obtained using:

(9) $$false-positive\;ratio\;\left[\%\right]=\frac{\sum_{i=0}^{T}{\left(\mathrm{number}\;\mathrm{of}\;\mathrm{good}\;\mathrm{nodes}\;\mathrm{classified}\;\mathrm{as}\;\mathrm{black}-\mathrm{hole}\;\mathrm{nodes}\;\right)}_{i}}{\mathrm{total}\;\mathrm{number}\;\mathrm{of}\;\mathrm{classifications}}\times 100$$

The false-positive ratio is the percentage of good nodes mistakenly classified as malicious by good nodes. Considering *G* as the total number of good nodes in the simulation and *BHs* as an abbreviation of black-hole nodes, the false-positive ratio for good nodes is obtained using:

(10) $$false-positive\;ratio\;for\;good\;nodes\;\left[\%\right]=\frac{\sum_{i=0}^{G}{\left(\mathrm{good}\;\mathrm{nodes}\;\mathrm{classified}\;\mathrm{as}\;\mathrm{BHs}\;\mathrm{by}\;\mathrm{good}\;\mathrm{nodes}\;\right)}_{i}}{\mathrm{total}\;\mathrm{number}\;\mathrm{of}\;\mathrm{classifications}\;\mathrm{by}\;\mathrm{good}\;\mathrm{nodes}}\times 100$$

## 4. Building the Reputation System

### 4.1. Approach to Building the Reputation System

To create a reputation system, various variables must be considered. The testing scenario has to be studied, the detection method chosen, and how to apply it is also vital. 

To choose the scenario, the first step was to select the number of nodes. Other important aspects of the scenario are the node movement model and the message generation rate. Although the number of messages created is an important variable, especially to evaluate a reputation system, it was decided that for the initial simulations, all nodes would create messages at the same rate. There are more factors involved in the simulation scenario, but these were the main factors chosen to be evaluated at a specific level. 

With the simulation scenario set, the reputation system began to be created. The system’s creation was divided into two parts: The detection phase and the action phase. The detection phase, as the name suggests, addresses the detection of black-hole nodes by nodes in the network. In turn, the action phase uses the detection made to punish the bad nodes.

### 4.2. Simulation Scenario

The ONE simulator [23] gives a plethora of options when it comes to the simulation scenario. The number of nodes in a network and its speed influence the number of contacts between nodes positively, and consequently, the number of messages transmitted. In the same size map, fewer nodes will lead to a sparser network, while more nodes will result in a denser network. With a sparse network, there are fewer contacts; therefore, there are few opportunities for exchanging messages.

Nevertheless, with fewer messages, node buffers are not as congested and can carry messages longer without dropping them. For dense networks, the exact opposite happens. With nodes moving faster, more contacts happen, but contacts are shorter, leading to fewer messages transferred in each contact. 

The overall simulation settings are presented in Table 2. The Helsinki downtown map was used similarly to [9,24]. To represent better the diversity that a DT-IoV network might have, three types of nodes were considered: Vehicles, cars, pedestrians, and trams. 

The routing protocol chosen for the analysis was Prophet as it is one of the most commonly used protocols. If a protocol uses none or very few intermediate nodes between a source and a destination, like the Direct Delivery [25] or Spray-and-Wait [26] protocols, identifying malicious nodes will be impossible or very slow. As a matter of fact, Direct Delivery is unaffected by black-hole nodes [8], as a source node only delivers a message if it meets the message destination node, thus not requiring any intermediate node, which makes it impossible to detect black-hole nodes. Naturally, the disadvantage of Direct Delivery is poor routing performance. Spray-and-Wait uses a very limited number of intermediate nodes, which slows down the detection of black-hole nodes. 

For all results presented, ten simulations were made for each scenario with different seeds for generating different node movement and traffic generation patterns to guarantee nondeterministic results in each run. The corresponding 95% confidence intervals are presented with the results. 

### 4.3. Detection Phase

#### 4.3.1. Independent Detection Scheme: Scheme Description

The detection part of the reputation system in each node has the purpose of identifying black-hole nodes in the scenario. For this reason, to classify the detection schemes, only three metrics were used: The detection ratio, false-positive ratio, and false-negative ratio.

Various strategies can be used to identify malicious nodes, as seen in the Background section. For this work, some considerations were deemed necessary. First, the detection phase must be decentralized, allowing each node to have its reputation rating for other nodes in the network. Second, the reputation rating should be achieved in the most independent manner possible, and when necessary, exchanged information between nodes should be done carefully. In addition, it is of higher importance to achieve the best metric possible and a fast convergence rate in the false-positive ratio than in the false-negative and the detection ratio. This decision was made because of the impact that the false-positive and false-negative ratios have in the action phase of the scheme. In a worst-case scenario where a reputation system has a detection ratio of 0%, the network’s impact would be approximately the same as if no reputation system was applied. The same goes for the false-negative ratio. If a system has a false-negative ratio of 100%, then all black-hole nodes are considered good, and it is the same scenario as if no reputation were applied. However, a high false-positive ratio classifies many good nodes as malicious. This is a bigger problem as the goal of the whole reputation system is to improve the delivery ratio of good nodes. Classifying good nodes as bad, considering that a punishment will be applied to these nodes will probably decrease the good nodes’ delivery ratio, which is the opposite to what is desired.

The first attempt at a detection scheme is straightforward. Each node has a black-hole node list and a good node list. The black-hole node list saves the identification of nodes that are classified as bad, and the good node list saves the identification of nodes that are classified as good. When a node receives a message, all nodes that contributed to forwarding the message, as listed in the path stored in the message, are classified as good, put on the good node list, and removed from the black-hole node list, if they were listed there. The message source is considered malicious and put on the black-hole list unless it is already on the good node list. This scheme is called the Independent Detection Scheme. A node is classified as bad if it is listed in the bad node list, and classified as good otherwise.

**Figure 1:**
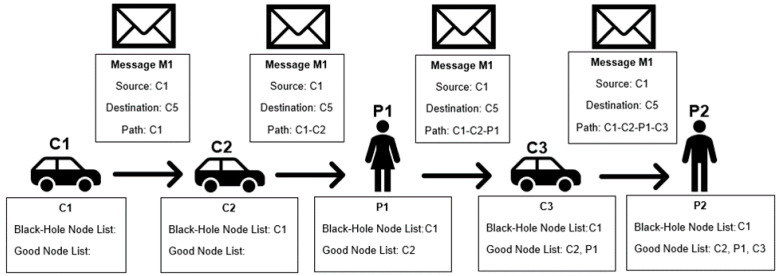
Independent Detection Scheme example.

**Figure 2:**
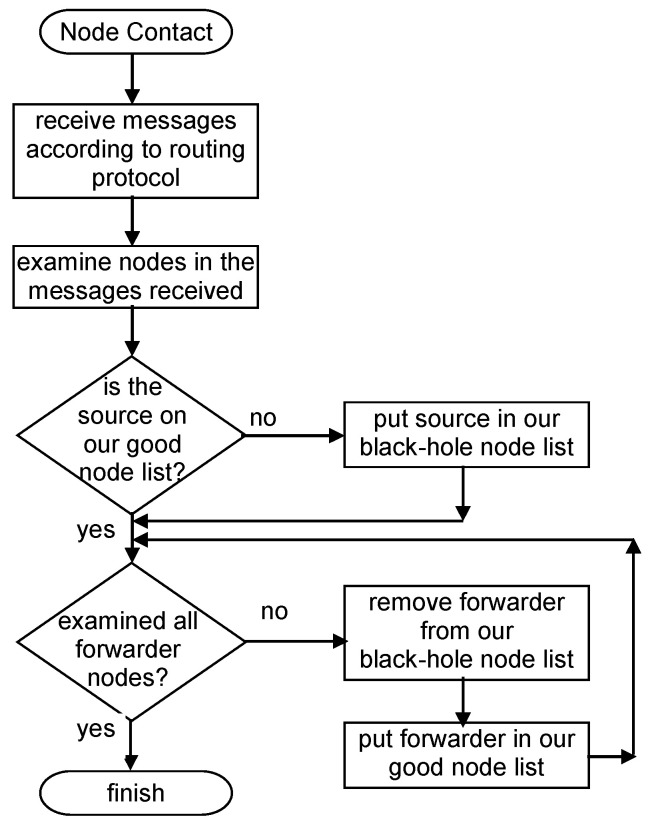
Independent Detection Scheme flowchart.

Figure 1 represents an example of the classification made in this detection scheme. In the figure, C refers to car and P to pedestrian; the following number serves as the identification of the node for scheme purposes. Figure 2 presents a flowchart of the detection scheme that is executed by each node when a contact is established with another node to exchange messages, detailing how the information in the messages received is used to update a node’s reputation tables. 

#### 4.3.2. Independent Detection Scheme: Analysis and Discussion

**Figure 3:**
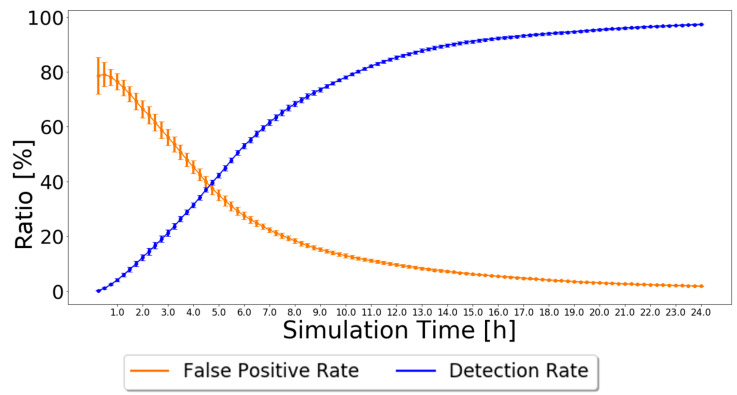
False-positive and detection ratios for Prophet with 20% malicious nodes for the Independent Detection Scheme.

As nodes’ behavior does not change during the simulation, every node that forwards a message in which it is not the source is good and will never change its status from the moment it is classified. Therefore, this detection scheme always leads to a 0% false-negative rate because not a single malicious node will be classified as good. The false-positive rate and detection rate have various results depending on the routing scheme used and the number of malicious nodes in the simulation. The results simulated in the ONE, for the settings presented in Table 2, are depicted in Figure 3 when using the Prophet routing protocol with 20% of black-hole nodes. The 95% confidence intervals are also plotted, showing great trust in the achieved results, particularly by the end of the simulation. 

The simulation results show that the false-positive ratio decreases to zero. Initially, nodes only have messages created by themselves. Thus, when nodes try to forward their messages, they will be considered malicious and added to the black-node list. Having 20% malicious nodes in the network results in about 20% being correctly classified as malicious and about 80% incorrectly classified as malicious. This is why the false-positive rate starts at about 80%. As good nodes forward messages, their classification will change to good, and the false-positive rate decreases, tending to zero after about 24 h. 

The simulation results also show that the detection ratio increases as time goes by. In the beginning, black-node lists and good node lists are empty, so the detection ratio accounts for zero black holes detected by every node. As nodes generate messages, they will be classified as malicious unless they contribute to the forwarding process, so the detection ratio gradually increases, reaching about 97% after 24 h.

If a modification is done such that a node is only classified as malicious after being the source of a message *n* times without forwarding messages, the detection ratio is much slower, and the false-positive ratio does not improve. 

In the independent detection scheme, every node classifies other nodes independently. Thus, the correct classification is not very fast. If nodes cooperate in the classification process, the classification can be faster. 

#### 4.3.3. Exchange Good Nodes Tables Detection Scheme: Scheme Description

Making nodes exchange reputation information should improve the speed of the classification mechanism. We propose the Exchange Good Nodes Tables Detection Scheme, where nodes send their good node list when they send a message to another node. This allows nodes to have information about other nodes they did not meet before without requiring any centralized server. To prevent accepting wrong information from malicious nodes, good node lists are used only if received from a node that has been classified as good, being deleted otherwise. 

**Figure 4:**
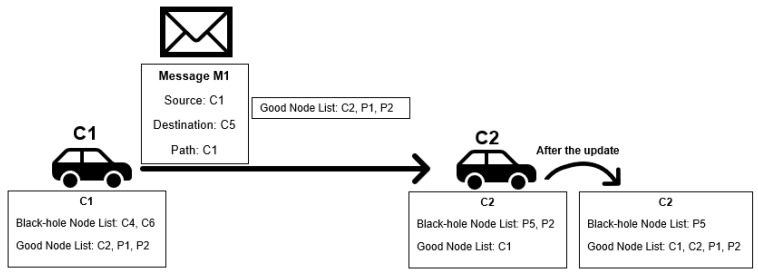
Black-hole and good-nodes lists update scheme example.

**Figure 5:**
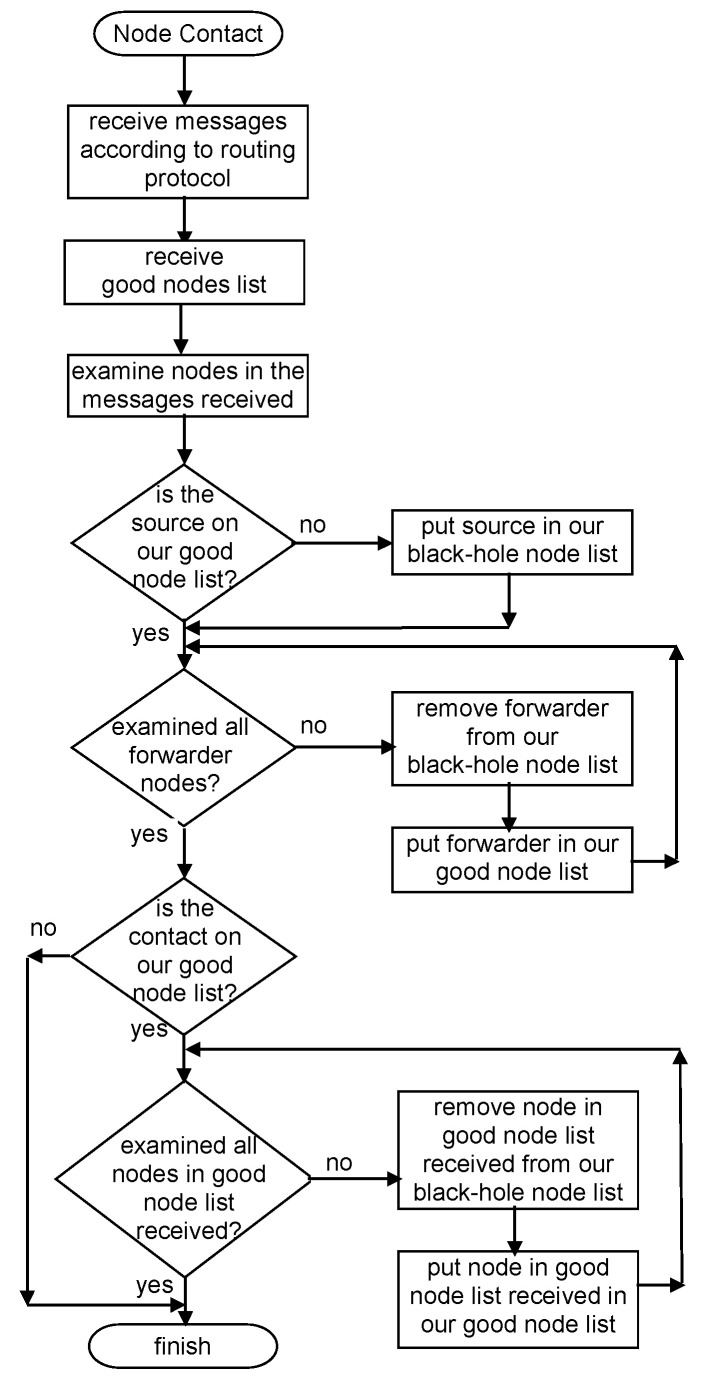
Exchange Good Nodes Tables Detection Scheme Flowchart.

This means that, as nodes can only classify other nodes as good if they have a message forwarding proof and this proof is always accurate, the information will always come from a good node, and its use will never be erroneous. The black-hole node list is not exchanged, as nodes have no proof that other nodes are black-holes. A picture exemplifying the Exchange Good Nodes Tables Detection Scheme is presented in Figure 4. Figure 5 presents a flowchart of the detection scheme that is executed by each node when a contact is established with another node to exchange messages and information in the reputation tables. 

#### 4.3.4. Exchange Good Nodes Tables Detection Scheme: Analysis and Discussion

**Figure 6:**
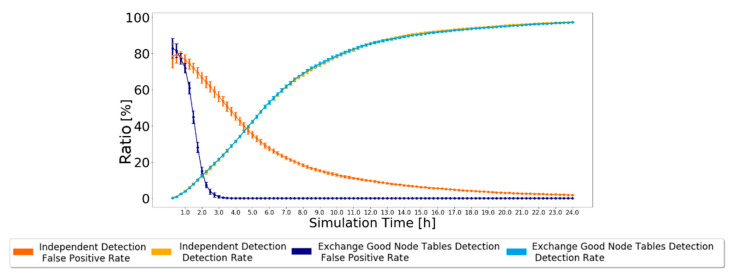
False-positive and detection ratio for Prophet with 20% malicious nodes for the Exchange Good Nodes Tables Detection Scheme 97. which is a very good result. However, now, the false-positive ratio decreases much faster as good nodes classifications are exchanged. The false-positive ratio was 0% as early as the fourth hour of the simulation, while in the Independent Detection Scheme, it had not reached 0% after 24 h of simulation.

The results simulated in the ONE, for the settings presented in Table 2, for Prophet with 20% of malicious nodes are presented in Figure 6, along with the results from the Independent Detection Scheme for comparison. The 95% confidence intervals are again very small, except for the simulation’s beginning, showing great trust in the achieved results. 

If good node lists received from nodes not yet classified as good are stored, waiting for the node to prove as good to be used requires significant additional memory and only marginally improves the classification performance. This is why good node lists received from nodes not classified as good are dropped. 

### 4.4. Action Phase

The action part of the reputation system in each node has the purpose of punishing black-hole nodes. The metrics used to check the reputation system’s performance were the delivery ratio for good nodes, the false-positive ratio for good nodes, and the detection ratio for good nodes.

For the action phase, the type of punishment selected and the time when nodes should start to apply the punishment are relevant. Considering the detection scheme chosen, if punishment is applied too soon, nodes might not have time to reach the false-positive ratio of 0%. By this, good nodes will be incorrectly penalized. On the other hand, if punishment is applied too late, it might not affect the nodes exchanges enough to change the delivery ratio of good nodes significantly. 

For punishment, three main and independent schemes were considered: An action related to creating messages, an action regarding the connection between nodes, and an action associated with deleting messages. Hereafter, they will be referred to as the Creation, Disconnect, and Delete action, defined as:

- Creation Action: Nodes do not create messages for nodes that they have in the black-hole node list.
- Disconnect Action: When a node is within range of another node that is in the black-hole node list, no connection is established, and no messages are exchanged.
- Delete Action: All messages from nodes in the black-hole list are deleted from buffers.

**Figure 7:**
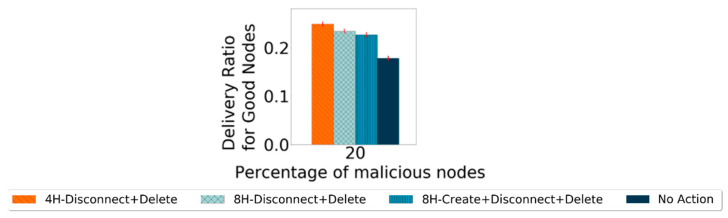
Comparing best performance action schemes’ delivery ratio for good nodes for Prophet with 20% malicious nodes.

These were the main actions taken to punish the black-hole nodes. As one should not assume which scheme will perform better or even if all actions together provide a better result, all possible combinations of actions were tested. To decide when to start applying the punishments, the results from the detection scheme were observed. Prophet reaches a false-positive ratio of 0% around the fourth hour of the simulation. For this reason and evaluation purposes, it was decided to test the actions scheme starting at the beginning, and at the second, fourth, and eighth hour of the simulation. The simulations use the defined settings in Table 2. In the first instance, for selecting the best options for the action scheme, only the metric of the delivery ratio for good nodes is evaluated. A case called “No Action” is used, representing the delivery ratio for good nodes if no action was taken to punish the black-hole nodes. From the results, the three action schemes with the best performance were chosen to be analyzed in more depth, and the corresponding results are presented in Figure 7 with 95% confidence intervals. 

As the most important part of a detection scheme is to increase the number of messages exchanged between good nodes, they should have a better delivery ratio for good nodes. The action scheme that provides the highest delivery ratio for good nodes more times is the 4H-Disconnect + Delete. However, this cannot be the only indicator to choose an action scheme, as it is still necessary to observe the action scheme’s impact on detecting nodes. It is expected that changes for either metric will only start appearing once the action scheme starts. If a false-positive ratio for good nodes of 0% is reached before the action phase starts, the action scheme should not have an impact on the false-positive ratio for the good nodes metric, whereas if it is not reached, the false-positive ratio will be higher than if no action was taken. This is mostly because, in any of the three action schemes, the Disconnect action prevents nodes from exchanging messages with nodes that they consider bad, making the detection process more difficult. For protocols in which the false-positive ratio of 0% is not reached, this metric will be higher the earlier the action scheme is applied. For the detection ratio for good nodes, it is also expected that the performance will decrease once the action takes place, as in any of the action schemes selected, there is a disconnect from the nodes that are considered malicious. Thus, no exchange of messages with malicious nodes will happen. The messages from nodes considered malicious no longer circulate on the network, which prevents some nodes from being identified as malicious, also preventing some nodes from proving themselves as good. 

**Figure 8:**
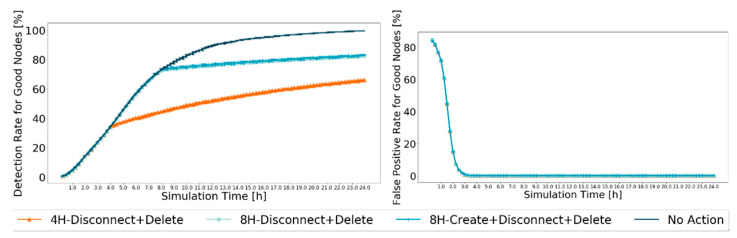
Comparison of detection ratio for good nodes and false-positive ratio for good nodes, between best performance action schemes, for Prophet, with 20% malicious nodes.

The results for these metrics were simulated using the defined settings in Table 2. The 95% confidence intervals are very small, showing great trust in the achieved results presented in Figure 8.

The results concur with those expected. The false-positive ratio for good nodes reaches zero before 4 h of the simulation, so it is not affected by the actions taken. However, when messages from black-hole nodes start being dropped, fewer messages circulate in the network, reducing the opportunities for good nodes to prove themselves as good, so the detection ratio for good nodes grows slower when traffic in the network reduces as a result of the action schemes. Furthermore, it does not appear to be a difference between the action schemes in which the action scheme starts at 8H. This can be because creating messages only for nodes that are considered good does not guarantee that those messages will be exchanged. Furthermore, it limits the messages created to only some good nodes, ending up excluding all nodes that are good but have not yet been identified as such. 

After analyzing the scenarios, the best combination of an action scheme is 8H-Disconnect + Delete. It is the second action scheme with the best delivery ratio for good nodes, not being outperformed by the 4H-Disconnect + Delete action scheme by a large value, but outperforming the 4H-Disconnect + Delete action scheme by a lot in terms of detection and false-positive ratios.

Due to how the nodes are classified, in a binary way, always being good or bad and not having any “status” in-between, the reputation scheme was named Binary Reputation System, or for short, BiRep. Naturally, nodes that are not yet in the good node list or black-hole node list are not yet classified, assumed as being good by default. 

## 5. Robustness Results and Analysis

In the previous sections, the results were studied to aid the development of the BiRep algorithm. This section has as the objective to test the reputation system against several different scenarios in order to determine BiRep’s robustness. To evaluate the results, only metrics for good nodes will be judged once they are most relevant to evaluate the whole scheme’s usefulness. 

As the most important factor for the success of a reputation system is the number of messages relayed between nodes, the first group of scenarios uses different message creation intervals and transmission rates to see how these differences influence the results. The second group of scenarios that are tested has a major focus on node density. 

### 5.1. Varying the Message Generation Rate

Most of the parameters for the scenarios are equal to the ones described in Table 2 of Section 4.2. Note that the “Base” scenario corresponds to the exact settings of Table 2 and serves as a comparison for the other scenarios. In the “Less messages” scenario, a node creates a message every 35–70 s. In the “More messages” scenario, each node generates messages in an interval from 9 to 18 s. The “Bigger transmission rate” scenario maintains the message generation rate from the base scenario, but the transmission rate from the nodes’ interfaces increases to double. This is summarized in Table 3.

**Figure 9:**
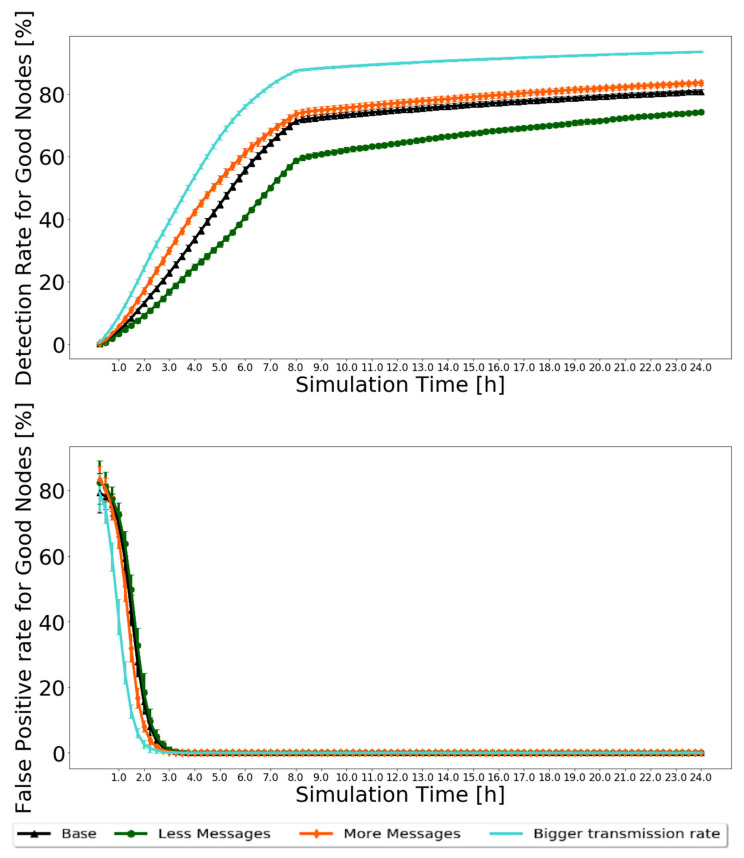
Comparison of detection ratio for good nodes and false-positive ratio for good nodes for Prophet, with 20% of malicious nodes to test the robustness of scenarios with different message generation and transmission settings.

First, looking at the node classification metrics, it would be expected that, for the “Less messages” scenario, fewer black-hole nodes would be identified, and for the “More messages” and “Bigger transmission rate” scenarios, more. For the “Less messages” scenario, fewer messages would be created. Therefore, fewer messages circulate in the network, and fewer opportunities exist for nodes to prove themselves as good. The exact opposite happens with the “More messages” scenario. In the “Bigger transmission rate” scenario, the message transmission rate is double, allowing nodes to exchange more messages and having more and faster information to classify nodes as good or as bad. The results for the Prophet are presented in Figure 9 for 20% of malicious nodes. The 95% confidence intervals are very small, showing great trust in the results. 

**Figure 10:**
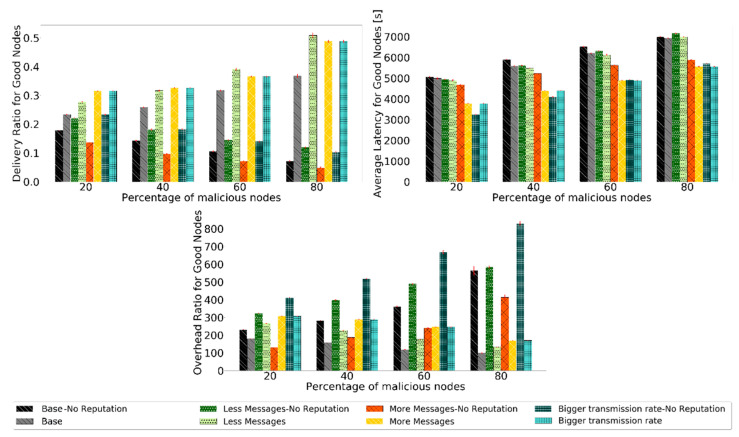
Comparison of delivery ratio for good nodes, overhead ratio for good nodes, and average latency for good nodes for Prophet to test the robustness of scenarios with different message generation and transmission settings.

Analyzing the results, the expected is confirmed. The detection ratio for good nodes increases in all scenarios, ranging from 70% to 90%, depending on the scenario. After 8 h, when the action phase starts and messages from black-hole nodes are dropped, traffic in the network is reduced, so the detection rate increases more slowly. The false-positive ratio decreases to zero for most of the simulation before 4 h. For the routing protocol metrics, it was decided to test the reputation system with various percentages of malicious nodes to test how BiRep would respond. The results for the same sets of simulations used for Figure 9 are presented in Figure 10. Again, the 95% confidence intervals are small, showing good confidence in the results. 

Without a reputation scheme, the delivery ratio, average latency, and overhead ratio for good nodes performance decrease as the percentage of black-hole nodes increases. Having more black-hole nodes in the network makes it harder for good nodes to deliver messages, resulting in a lower delivery ratio. When they deliver the messages, it takes longer, so more latency and, in general, more hops. The overhead ratio is also bigger because, with the increase in malicious nodes in the network, the same number of messages are relayed, but fewer are delivered using more hops. It is desired that all scenarios have a better performance than if no reputation scheme was used. 

Prophet, for all scenarios, shows better results for the delivery ratio for good nodes than if no reputation scheme was used. This proves the usefulness of the reputation scheme. Furthermore, the overall performance of the other metrics also improves. 

### 5.2. Varying the Node Density

The second group of scenarios is related to the type and quantity of nodes. The “50 nodes” and “106 nodes” scenario uses the simulation parameters presented in Table 2, except for the reduced number of nodes. The “All cars” scenario also uses the parameters of Table 2, except that there are 200 cars and 0 pedestrians. 

When the node density is modified, it is not easy to predict the results. While it is plausible to assume that 50 nodes will have fewer contacts than 106 or 206 nodes and thus fewer opportunities to exchange messages, the initial location and further movement of the nodes are random. Therefore, it is possible to end up with nodes moving very close to each other in the simulation, increasing the contact opportunities. Furthermore, with fewer nodes, fewer messages need to be exchanged before all malicious nodes are identified. Results depend immensely on nodes’ behavior and on the simulation scenario in general. For the scenario with all cars, it is not easy to predict results as well. Having more cars, and therefore more vehicles moving at a higher speed, increases the encounter possibility and can decrease the time available for message exchange. 

**Figure 11:**
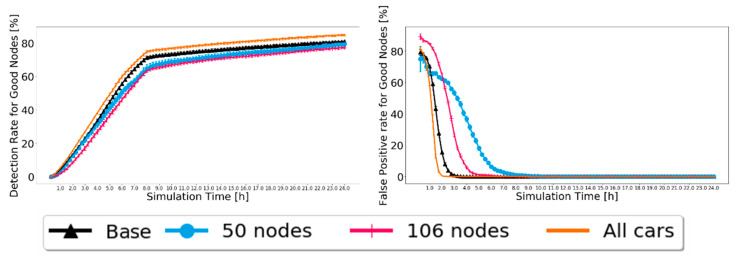
Comparison of detection ratio for good nodes and false-positive ratio for good nodes for Prophet, with 20% of malicious nodes to test the robustness of scenarios with different node numbers and profiles.

The results for the Prophet are presented in Figure 11 for 20% of malicious nodes. Again, the 95% confidence intervals are small, showing good confidence in the results. The detection and false-positive ratios behave as expected, reaching high detection levels and a 0% false-positive ratio. In addition, overall, a better detection ratio and faster convergence of the false-positive ratio to zero are related to a larger number of nodes, resulting in more contacts and, therefore, more message exchanges, thus having more and faster information. For the detection ratio, not a big difference is noted. This means that BiRep does not seem to be greatly affected by the profile or number of nodes. 

**Figure 12:**
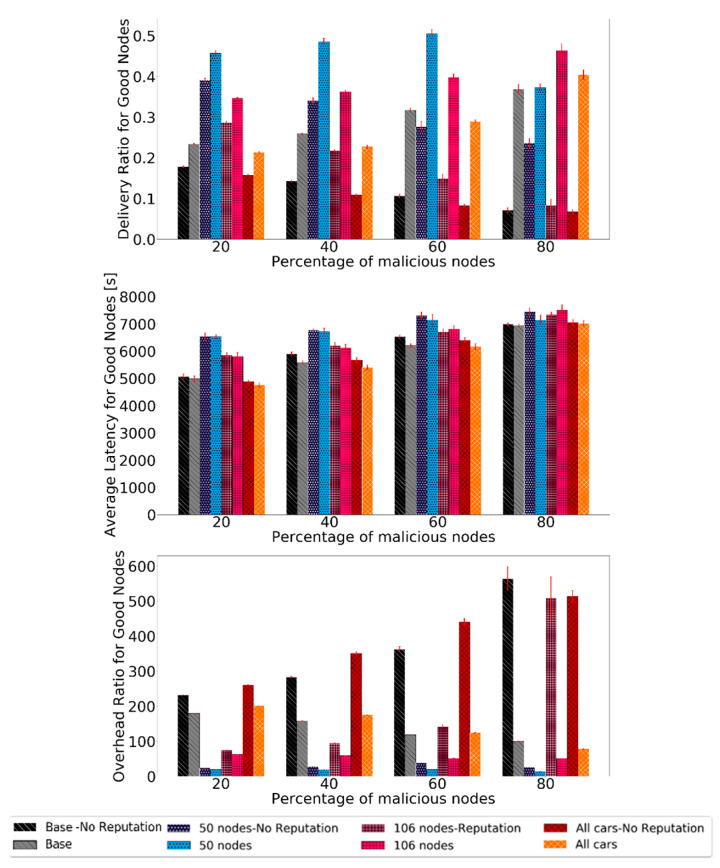
Comparison of delivery ratio for good nodes, overhead ratio for good nodes, and average latency for good nodes for Prophet to test the robustness of scenarios with different node numbers and profiles.

Evaluating the routing protocol metrics, the results are shown in Figure 12. The results show a big increase in the delivery ratio and overhead ratio for all scenarios. The latency does not change in such a significant matter, but it does not get worse either. 

Overall, BiRep performs very well in all scenarios. The delivery ratio can be, in some cases, even ten times larger than if no reputation is used. The latency is not significantly affected by the node density, speed, or message generation rate when reputation is used. The overhead ratio also presents significant gains as it can be reduced ten times by using the reputation mechanism. 

### 5.3. Comparing BiRep with the State-of-the-Art

Finally, we compare BiRep with other reputation schemes described in Section 2. For FBIDM [10], the overall conclusion was that for the Prophet routing protocol, the false-positive ratio was kept between 2% and 5%. The percentage of malicious nodes detected was around 80% at best. Although a straight comparison cannot be made, as neither the simulator nor the simulation parameters are the same, the scenario with 50 cars is similar enough to try and make a fair comparison. Figure 11 shows that for the Prophet protocol, the detection ratio is set around 70–80% and 0% for the false-positive ratio. The results are not much different, but BiRep has the benefit of not having a single point of failure, as it is not a centralized system, and nodes can make decisions by themselves. 

For MUTON [13], the simulation results are better: 1–2% false-positive ratio and 95% detection ratio. This scheme has 10–30% of malicious nodes in the simulation. For that scenario, again comparing with the 50 nodes scenario for 20% of malicious nodes, BiRep has a 0% false-positive ratio and at least 80% detection ratio. MUTON has a better detection ratio but problems associated with having a centralized detection scheme. 

**Figure 13:**
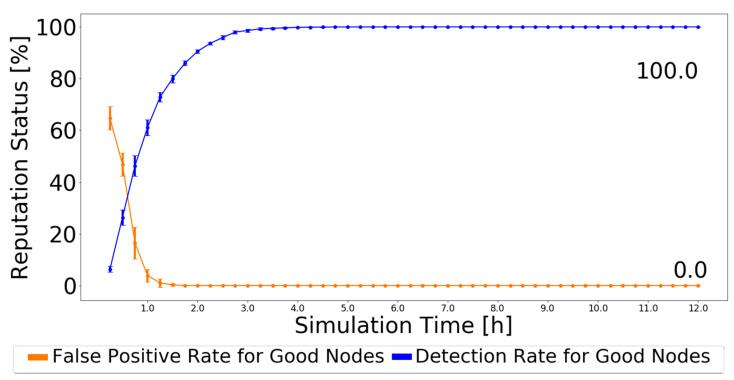
Detection ratio for good nodes and false-positive ratio for good nodes for Prophet with simulation settings of [14] and BiRep.

Compared with the MDS in [14], for Prophet, the MDS scheme has a detection ratio higher than 90%, reaching 97%, and 0% false-positives. To make a fair comparison, as the simulator used in [14] is the same in this work, and the authors provided the simulation settings, a test was made. The detection scheme of BiRep was applied but using the simulation settings of [14]. Actions started being applied at 10,000 s, as in [14], and using the Disconnect Action scheme. The results are presented in Figure 13. 

As the simulation results prove, at least the detection scheme part of BiRep works very well, outperforming the results of MDS [14] for black-hole nodes, and for the most part, reaching a perfect result in terms of detection. 

For [16], again, only the Prophet protocol is analyzed. A fair comparison cannot be made, as the simulator and movement models are not the same. The detection rate for different mobility schemes is 85–100% and the false-positive rate is around 2%, in the best case. The results, in general, are worse than those for BiRep. When comparing, BiRep does stand out as a good option. However, to make a completely fair comparison, further simulations should be made. That would require access to the other works’ source code, so it is left for further work. 

Our solution also assumes that nodes do not change behavior, so there are no grey-hole nodes and nodes do not collude. Even when looking only at a scenario where black-hole nodes do not change behavior and do not collude, there are still flaws. If direct delivery were to be used, all nodes would be considered malicious as they are always the source of the messages they carried. Nevertheless, this can also be considered a nonproblem as Direct Delivery performance is not affected by black-hole nodes. Table 4 presents a comparison between BiRep and state-of-the-art schemes regarding false positive and detection ratios.

## 6. Conclusions

This article’s main objective was to develop an effective decentralized reputation scheme for DT-IoV, adaptable to various network scenarios to diminish black-hole nodes’ effects in the network. In the detection phase, the Exchange Good Nodes Tables detection scheme was selected as the best choice as it is decentralized, requires little memory in the nodes, and can classify nodes without encountering them. For the Action Phase, selecting the best action was to disconnect from the identified black-hole node and drop messages from black-hole nodes after eight hours, which is long enough to have a 0% false-positive in most scenarios, and avoid excluding good nodes from the network. However, for routing protocols where messages have a very small number of hops, like Spray-and-Wait, it may take much longer to identify malicious nodes correctly.

BiRep offers a solution to deal with black-hole nodes that significantly improve routing protocol metrics. The simulation results for different scenarios prove BiRep’s versatility, achieving in all scenarios an up-to-10 times improvement in delivery ratio and overhead ratio, helping to increase the performance of the overall network.

Some ideas are left for future work: Studying the case of collusion attacks; using social information to improve the classification of nodes; using adaptive mechanisms in the action phase to deal with protocols with a reduced number of hops like Spray-and-Wait, and to deal with node redemption; investigate the use of the Blockchain technology to maintain reputation information as in [27].

## Figures and Tables

**Table 1:** Delay-tolerant networking (DTN) black-hole (BH) detection and mitigation schemes-related work.

Scheme	Information Used	Detection	Action	Limitations
FBIDM [10]	past meetings, past delivery probability	suspicious multiple times	exclude black-holes	requires ferry nodes
MUTON [13]	past meetings, past delivery probability, transitivity	suspicious multiple times	exclude black-holes	requires ferry nodes
MDS [14]	encounter records	forwards less than a threshold	exclude black-holes	no exchange of trust information
MDS extension [15]	encounter records, cluster analysis	forwards less than a threshold	exclude black-holes	no exchange of trust information
Packet Exchange Recordings [16]	delivery records	forwards less than a threshold multiple times	none described	no exchange of trust information
RCAR [17]	messages carry forwarders, ACK messages	aging decreases reputation	prefer nodes with a higher reputation	no exchange of trust information, requires ACK
CWS [18]	messages delivered, relayed, dropped	exchange of reputation, thresholds	few resources used for nodes with low reputation	does not assess classification performance
BiRep	messages carry forwarders	exchange of reputation, node has no forwarding record	warmup, disconnect from black-holes, delete messages from black-holes	

**Table 2:** Simulation parameters.

Simulation Time	24 h
Map	Helsinki downtown (4500 m × 3500 m)
Movement Model	Shortest Path Map-Based Movement Model
Nodes’ speed	Pedestrians 1.8–5.4 km/h; Cars 10–50 km/h; Trams 25–36 km/h
Number of nodes	206 (Pedestrians: 100; Cars: 100; Trams: 6)
Nodes’ buffer size	5 MB
Nodes’ wait time	0–120 s
Message size	500 kb–1 MB
Message generation interval	25–35 s
Message TTL (Time to Live)	5 h
Interfaces’ data rate	250 kBps = 2 Mbps
Interfaces’ transmission range	10 m

**Table 3:** Settings assigned to different simulation scenarios to assess message generation impact.

Scenario Name	Base	Less Messages	More Messages	Bigger Transmission Rate
Message generation interval	25–35 s	35–70 s	9–18 s	25–35 s
Interfaces’ data rate	2 Mbps	2 Mbps	2 Mbps	4 Mbps

**Table 4:** Comparison of BiRep with the state-of-the-art regarding false positive and detection ratios.

Reputation Schemes	False Positive Ratio	Detection Ratio
FBIDM	5%	80%
MUTON	2%	95%
BiRep	0%	80%
